# Contextual deep learning for accurate news article categorisation with pre-trained embeddings

**DOI:** 10.1038/s41598-026-38998-3

**Published:** 2026-04-18

**Authors:** Ameer Hamza, Asif Muhammad, Muhammad Sohail Abbas, Sana Ullah Jan

**Affiliations:** 1 National University of Computer & Emerging Sciences (NUCES), Islamabad, Pakistan; 2 School of Computing, Engineering, and the Built Environment, Edinburgh Napier University, UK

**Keywords:** Engineering, Mathematics and computing

## Abstract

An increasing amount of online news content in digital journalism leads to novel and complicated issues regarding its classification and organisation. Systems that automate operational tasks can provide numerous advantages over systems that manually classify documents. The current work attempts to solve this problem using contextual and semantic deep learning. The text’s semantic meaning is captured using pre-trained word embeddings, and understanding is aided by a hybrid neural structure that incorporates local and distant text dependencies. This technique is compared against classical machine learning on two prominent news datasets. The deep learning approach yields a remarkable improvement in classification, reaching over 91% accuracy on the AG News corpus, a balanced four-class English benchmark, while its performance on the News Category Dataset V3, which is more complex and highly unbalanced, is considerably lower. These results highlight the effectiveness of contextual and semantic modelling for news categorisation, while also illustrating the impact of dataset complexity and class imbalance on achievable performance.

## Introduction

The advancement of digital journalism has also made available a vast array of online articles covering numerous topics and written in diverse styles and languages. The quantity of these articles has outstripped the ability of practitioners to perform manual sorting, thus demonstrating the need for automated, intelligent and scalable sorting. The challenges of classifying news articles dealt with the analysis of various lexicons and the semantics of context, an area where rule-based algorithms and shallow learning approaches fail. The application of NLP and machine learning in recent years has increased the scope of research in the area of semantic-aware text classification.

The application of traditional machine learning methods, such as Random Forest, Naïve Bayes, and Support Vector Machines, to text classification problems has been extensive, though they are primarily limited to the news domain because of the aforementioned challenges. The main constraint they exhibit is the inability to identify the contextual linkages and the scope of ambiguities, as well as the numerous definitions in news content. These models are heavily reliant on superficial lexical structures, and thus, they are unable to cope with contextual variations and the abstract narratives which are so prevalent in news articles. Moreover, they lack robust mechanisms to model polysemy and fail to preserve the sequential structure of textual information, which is crucial for understanding semantic intent. Given the high degree of category overlap, contextual variability, and the growing demand for interpretability, the efficiency of traditional machine learning models for real-world news classification remains constrained.

Deep learning models have demonstrated superior capability in modelling both local and global textual patterns, as demonstrated in prior work on CNN-based sentence modelling^1^ and hierarchical document-level architectures^2^. Convolutional Neural Networks (CNNs) have been shown to effectively capture local n-gram features and salient phrase-level patterns, while hierarchical and sequential architectures can model long-range document-level dependencies and contextual relationships. More recently, transformer-based architectures leveraging self-attention mechanisms have enabled deep contextual language understanding by jointly attending to all token positions, significantly improving performance across a wide range of NLP tasks^3^. In this study, a deep learning-based news classification framework is proposed that integrates convolutional and recurrent neural architectures alongside pre-trained semantic embeddings to address these challenges. Recent applied studies in domains such as financial forecasting have further emphasised the necessity of systematic comparative evaluation of deep learning architectures to balance performance, stability, and practical deployment feasibility^4,5^. Motivated by this, the proposed architecture integrates global semantic word representations for meaning encoding and uses a hybrid neural framework for the local textual pattern capture and the long-range context pattern capture. The architecture attempts to perform feature extraction and sequence modelling by CNNs to achieve simultaneous feature learning and contextual analysis, and to adapt to the complex structure of news articles. A contextual machine learning baseline is provided for comparative analysis to illustrate the benefits of the proposed context-sensitive deep learning framework.

This study summarises the primary contributions of this research. In this case, there is no new learning technique or change in architecture. Instead, this study offers a systematic empirical investigation of a contextual learning framework based on CNN and LSTM for news categorisation in the context of both balanced and imbalanced data. The research analyses a hybrid classification framework that integrates spatial and sequential modelling in text using semantic embeddings. Under both balanced and imbalanced settings, the framework is benchmarked on two real-world news datasets, namely the News Category Dataset V3 and the AG News Corpus. Comparative experiments are conducted against a contextual machine learning baseline to analyse performance in terms of accuracy, recall, and F1-score. The contribution of this study lies in providing reproducible insights into the behaviour, strengths, and limitations of contextual deep learning models when applied to real-world news datasets of varying complexity. In addition, the findings highlight the role of contextual modelling in text classification and establish a foundation for future work on improving transparency and interpretability through attention mechanisms and Explainable AI (XAI) techniques.

The remainder of the paper is organised as follows. Section “Literature review” provides a detailed review of related work in news classification using both ML and DL techniques. Section “Proposed methodology” describes the proposed methodology, including preprocessing, embedding, and model design. Section “Experimental setup” explains the evaluation metrics employed to assess model performance. Section “Results analysis” presents the experimental setup, datasets, and results under different conditions. Section “Conclusion” offers a comprehensive analysis of the comparative findings, summarises the paper and discusses potential directions for future research.

## Literature review

The use of automated news generation paired with social media has skyrocketed, leading to more unstructured text, which requires more sophisticated automated text classification systems. There is a certain simplicity and straightforwardness to older classification systems, which makes traditional machine learning algorithms appropriate. In this regard, the Random Forest (RF) algorithm is particularly interesting because of its classification performance with texts and its ability to overcome the problem of overfitting because of the model averaging from its multiple decision trees. RF has been successfully applied to various classification problems within the domain of news, and with constructed features such as TF-IDF and n-grams. For instance, as part of the BNeC Corpus, Barua et al.^6^ applied RF along with some other machine learning algorithms to classify sports news in Bengali, and reported encouraging results using trigram and unigram frequencies.

Zeeshan et al.^7^ have also suggested a Context-Aware Text Classification (CATC) system, which increases the performance of RF through advanced preprocessing techniques such as stemming or removing stop words. These studies pointed out the strengths and the limitations of RF; while robust, it struggles with the semantics and the more complex sequence-dependent elements. However, with the advancement of language, particularly in the context of news, the limitations of static features, fixed RF classifiers, or classifiers with fixed semantic models have become apparent.

CNNs have emerged as a strong baseline for text classification tasks. Kim^1^ demonstrated that shallow CNN architectures using multiple filter widths effectively capture local n-gram patterns in sentences and documents, achieving competitive results across multiple NLP benchmarks. As a result, CNNs have become a standard local feature extractor in news and document classification. Elements such as polysemy, jargon, and long-distance dependencies frustrate the more nuanced classifications of RF models. For example, Petukhova and Fachada^8^ concluded that the traditionally constructed models, which support hierarchical and multilabel classification, do not work, highlighting the necessity for more abstract semantic models. In addition, RF cannot explicitly model temporal structures, which excludes it from tasks in which the order and the flow of words are crucial to meaning.

To capture document-level context beyond local patterns, hierarchical architectures have been proposed. Yang et al.^2^ introduced a Hierarchical Attention Network (HAN) that models text at both word and sentence levels, enabling the capture of long-range dependencies and global document semantics. Such approaches demonstrate the importance of structured contextual modelling in news classification.

Recently developed hybrid architectures with CNNs and Long Short-Term Memory (LSTM) networks have become predominant in deep learning for text classification. While CNNs successfully capture the spatial hierarchies of features, including the more localised n-grams, LSTMs are designed to capture the sequential and contextual relations of the text over a given length of passage. The hybrid CAP LSTMs then provide a more complete framework for the retrieval of both the local and global semantic features necessary to understand a passage. The effectiveness of combining local and global feature representations has been empirically validated by Javed et al.^9^, who demonstrated that hybrid architectures integrating local feature extractors with global sequential or modelling components yield improved robustness and representational capacity. This finding directly supports the architectural choice of integrating CNN-based feature extraction with recurrent modelling for news text classification.

Empirical evidence further supports the effectiveness of CNN+LSTM hybrids. Zhai et al.^10^ employed a multi-scale CNN+LSTM architecture and achieved a validation accuracy of 93.41% on news datasets. In the same way, Umer et al.^11^ used CNN structures and FastText embeddings for the AG News Corpus and Yelp datasets, and in word order and meaning structure critical situations, they reported gains in both accuracy and F1 score against traditional baselines.

Extensions of the CNN+LSTM architecture that incorporate the pretrained word embeddings GloVe and Word2Vec have improved the learning of semantic representation. These embeddings encode individual words as vectors in a dense space for which the distance corresponds to semantic similarity, capturing contextual meaning. Ilie et al.^12^ evaluated multiple embedding strategies in misinformation detection and found that Word2Vec and GloVe embeddings significantly improved CNN and RNN performance. Likewise, Liu^13^ and Li et al.^14^ using CNN+LSTM and attention-based architectures reported classification accuracy of above 94% on news datasets while stressing the importance of semantic embeddings and sequential modelling.

Despite these advances, CNN+LSTM models present notable limitations. Training such architectures requires substantial computational resources and large volumes of labelled data. Furthermore, deep learning models are often criticised for their lack of interpretability, raising concerns in sensitive domains such as journalism and political reporting. To mitigate these issues, XAI techniques such as SHAP and LIME have been explored. Tabassoum and Akber^15^ investigated these methods for interpreting CNN-based news classifiers, demonstrating their potential to enhance transparency.

Transformer-based models have redefined contextual representation learning in NLP. Devlin et al.^3^ introduced BERT, which leverages bidirectional self-attention to learn deep contextual embeddings and has become a dominant benchmark for text classification, including news categorisation. In comparative studies, transformer-based models like BERT and RoBERTa have shown superior performance due to their contextual embedding capabilities. Still, their computational demands limit their use in resource-constrained environments. Eduardo et al.^16^ and Parvathavarthini et al.^17^ demonstrated that while BERT outperforms traditional ML models, its inference time and hardware requirements pose challenges for real-time or large-scale deployment. In contrast, CNN+LSTM with pretrained embeddings offers a compelling trade-off between performance, efficiency, and contextual understanding, especially when applied to medium-sized datasets like the News Category Dataset V3 and AG News Corpus.

Over the years, advances in deep learning, attention mechanisms, and transformer-based frameworks have driven substantial progress in text classification and news categorisation. Despite these developments, challenges remain in effectively capturing semantic nuance, handling polysemy, and addressing class imbalance, particularly in large-scale and fine-grained news datasets. To mitigate these issues, prior research has explored specialised embeddings, hybrid neural architectures, and data augmentation strategies, including approaches inspired by large language models. Guo et al.^18^ proposed the Polyseme-Aware Vector Representation Model (PAVRM), which integrates two complementary components: PAVRM-Context, where word embeddings are learned based on contextual usage, and PAVRM-Centre, which represents polysemous words through conceptual centroids derived from their contextual instances. Experimental evaluation on the SST2, R8, and R52 datasets demonstrated notable improvements in F1-score, precision, and recall for polysemous word disambiguation. However, the reliance on context clustering introduces substantial computational overhead, limiting the scalability of the approach for broader text classification tasks.

Mao et al.^19^ introduced a multi-level semantic feature extraction framework to enhance Chinese news classification. Extended TF-IDF and CHI algorithms were used for keyword extraction, TextCNN captured local semantic features, and BiLSTM with attention modelled global dependencies. Tested on THUCNews, LTNews, and MCNews datasets, the model outperformed a BERT+FC baseline by 1.2–2.8% in accuracy while maintaining low parameter complexity. Yet, risks of overfitting and restricted multilingual adaptability remained unaddressed.

**Table 1:** Summary of literature review on random forest and CNN+LSTM for news classification.

**Author & Publication Year**	**Problem addressed**	**Methodology**	**Results**	**Limitations**
Barua et al., 2021^6^	News classification using ML models	TF-IDF features with RF, SVM, LR	High F1-score using trigrams and RF	Limited contextual modeling; dataset-specific evaluation
Zeeshan et al., 2023^7^	Improve traditional ML through preprocessing	Tokenisation, stemming, stopword removal + RF	Enhanced RF performance	Increased complexity; lacks deep semantic capture
Petukhova & Fachada, 2023^8^	Hierarchical, multilabel classification	Multi-level dataset; ML model comparison	Validated semantic complexity	Traditional models struggle with semantic depth
Zhai et al., 2023^10^	Improve accuracy via hybrid DL models	Multi-scale CNN+LSTM with word embeddings	93.41% validation accuracy	Model complexity and high computational load
Umer et al., 2023^11^	Use word embeddings for richer semantics	CNN + FastText embeddings	Improved accuracy and F1-scores	High training resource requirements
Ilie et al., 2021^12^	Compare embeddings in DL models	CNN/RNN + GloVe, Word2Vec	Word2Vec + CNN performed best	Model overfitting and embedding sensitivity
Chen Liu, 2024^13^	Enhance deep semantic modelling in news text	CNN+LSTM with attention mechanism	Over 94% accuracy	Dataset-specific evaluation; limited scalability analysis
Xiaoyu Li et al., 2024^14^	Leverage attention in CNN+LSTM for text	CNN+LSTM with attention and GloVe embeddings	96.3% accuracy	High computation and training data requirements
Tabassoum & Akber, 2024^15^	Improve interpretability of RF classifiers	RF + SBERT embeddings + LIME/SHAP	91.48% accuracy; enhanced transparency	Complex interpretability for non-technical users
Eduardo et al., 2023^16^	Evaluate transformer vs classical classifiers	BERT vs traditional ML across corpora	BERT outperformed RF	High inference cost and hardware dependency
Parvathavarthini et al., 2023^17^	Large-scale news classification	Fine-tuned BERT with labelled data	91% accuracy (42 categories)	High computational requirements
Sakor et al., 2022^20^	Detect related posts using knowledge graphs	Graph-based semantic linking via ontologies	High accuracy in domain-specific settings	Domain-specific; limited scalability
Swati et al., 2023^21^	Predict bias using commonsense reasoning	COMET + ATOMIC2020 + classifier pipeline	Improved accuracy for political headlines	Dependent on external knowledge quality
Khudair et al., 2025^22^	Improve classification using deep features	CNN+GRU with Word2Vec embeddings	97.73% accuracy	No attention mechanism; potential overfitting
Kumar et al., 2025^5^	Assess the reliability and stability of deep learning models	Comparative evaluation of DL architectures under real-world conditions	Highlighted trade-offs between accuracy, stability, and deployment cost	Focused on applied settings; not specialised for the news domain

Hybrid architectures combining convolutional and recurrent layers have also shown promise. Zhai et al.^10^ introduced a multi-scale CNN+LSTM model, leveraging Word2Vec embeddings to capture local sentence structures and long-term dependencies. Achieving 99% training accuracy and 93.41% validation accuracy, it outperformed LSTM, TextCNN, and CNN baselines, although its complexity may hinder real-time deployment. Similarly, Zhu et al.^23^ improved semantic modelling for Chinese news by integrating ERNIE embeddings with a self-attention mechanism, a Deep Pyramid CNN, and BiGRU with soft attention, reaching 94.92% accuracy on THUCNews. While effective, such architectures demand substantial computational resources.

Transformer-based models have also been extensively explored for text classification. Garrido et al.^16^ conducted a comparative study of BERT and classical classifiers such as Logistic Regression and Support Vector Machines using TF-IDF features across multiple datasets, including news corpora. Their results showed that BERT achieved 90.93% accuracy on Portuguese news classification, outperforming AutoML predictors, which reached 84.80%. Despite these gains, the high computational cost and inference latency associated with transformer-based models continue to restrict their applicability in low-resource or large-scale settings. For short-text classification, Chawla et al.^24^ reported an accuracy of 81.47% using LightGBM with TF-IDF-weighted FastText embeddings, demonstrating that classical models combined with efficient feature representations can remain competitive under constrained computational budgets.

Using hybrid modelling techniques, Khudair et al.^22^ obtained 98.73% accuracy on the THUCNews dataset employing a CNN+GRU model with Word2Vec embeddings. The architecture where CNN layers capture short-range local contextual features while the GRU units model the long-range global semantic dependencies performed better than the bases of CNN, GRU, LSTM, and CNN+LSTM. Although the absence of attention mechanisms may increase susceptibility to overfitting, the model compensates through lower architectural complexity and robust semantic representation. The authors suggest that future work could incorporate attention mechanisms and refined hyperparameter tuning to further enhance performance.

More recently, hybrid models incorporating graph-based semantic augmentation and commonsense reasoning have been proposed to enhance contextual sensitivity in classification tasks. Sakor et al.^20^ introduced the PINYON framework, which leverages knowledge graphs and auxiliary textual domains to identify semantically related social media content. Similarly, Swati et al.^21^ proposed the IC-BAIT framework, which integrates commonsense knowledge bases such as COMET and ATOMIC2020 to support political bias reasoning. While these approaches demonstrate improved semantic depth, they are often computationally expensive and tailored to specific application domains.

In summary, as outlined in Table 1, although Random Forest models remain semantically shallow and sequence-agnostic, they continue to serve as efficient and interpretable baselines for text classification. However, the complexity of modern news articles necessitates deeper semantic understanding and sequence-aware modelling. In contrast, CNN+LSTM architectures enhanced with pretrained embeddings such as GloVe offer effective mechanisms for capturing complex semantico-syntactic patterns. Rather than proposing architectural innovations, this work adopts an established CNN+LSTM framework and evaluates it in a controlled and comparative setting, with particular emphasis on class imbalance and label granularity. In doing so, the study aims to balance classification performance, contextual sensitivity, and practical deployability.

## Proposed methodology

**Fig. 1:**
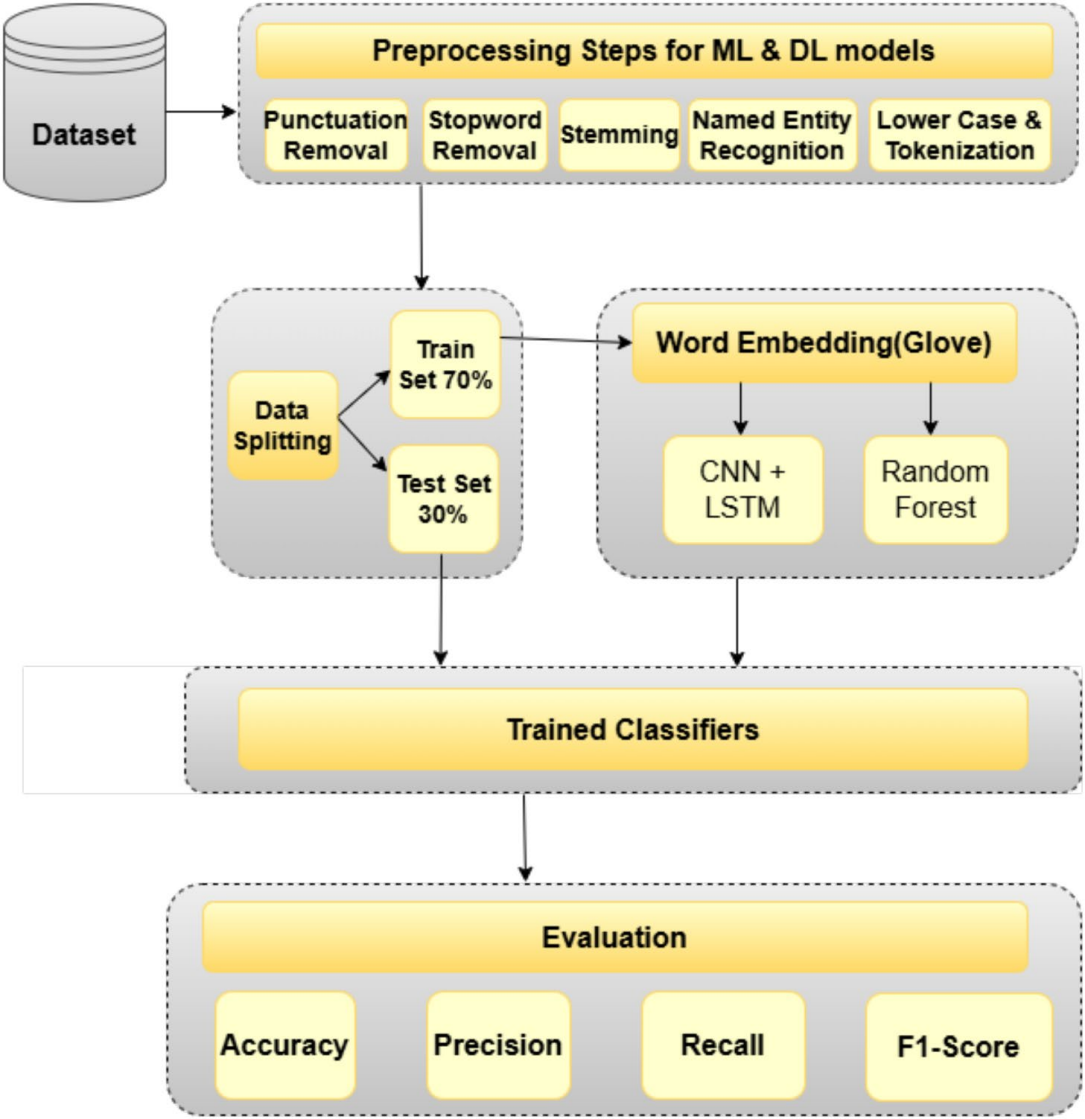
Proposed methodology illustrating preprocessing, data splitting, word embedding (GloVe), and dual model training using CNN+LSTM and Random Forest, followed by performance evaluation using standard metrics.

This section describes how a context-aware news classification system that employs high-level Natural Language Processing Techniques and neural networks is built. The framework employs classical and deep learning approaches within a context-embedding framework for enhanced Performance. The methodology comprises six phases as depicted in Fig. 1: data preprocessing, data splitting, word embedding, model design, training & classification, and evaluation.

### Dataset acquisition

The first step of the methodology consists of gathering a comprehensive and field-relevant news dataset. This comprises news articles from different sources and different varieties, including politics, sports, technology, entertainment, and world news. This news articles dataset acts as the base corpus for all subsequent processing, modelling, and evaluations. As an additional advantage, these news datasets can also be sent to the large language models for dynamic paradigm performance evaluation on an ensemble basis.

### Preprocessing pipeline

The different components of the preprocessing pipeline serves the purpose of standardisation and enhancement of the input data. This noise-reduction process includes the removal of punctuation and stopwords, the unification of word variants through stemming, and the application of NER, which was applied only for exploratory analysis and not used as an explicit model feature. The text is also lowercased for uniformity and then otherwise tokenised to produce individual words. The result is a clean, semantically enriched text that is ready for vectorisation and subsequent ingestion by the model.

### Data splitting

The original dataset was divided into three mutually exclusive subsets: training, validation, and test sets. The training set was used to learn model parameters, while the validation set supported hyperparameter tuning, early stopping, and model selection. The test set was separated for the exclusive purpose of evaluating the model’s performance and did not participate in the training and validation of the model at any point. This separation will help lessen the model training overfitting and provide an unbiased evaluation of the final model.

### Semantic embedding via GloVe

The subsequent step is utilising GloVe to transform tokens into vectors in order to obtain the structure of the text data. GloVe is a classical model of word representation that constructs a word embedding from count-based global word co-occurrence matrices in extensive text corpora and estimates the importance of a word. The embedding GloVe provides expresses the relationships of words in the proper context, owing to their semantic similarity. Similar vectors represent words used in the same context. These representations are then supplied to other deep learning models for further processing as primary, accurate contextual input.

### Classification models

Two distinct classification architectures are explored in this study to investigate the effectiveness of contextual and semantic modelling:

#### CNN+LSTM hybrid model

- CNNs are employed as local feature detectors that capture salient n-gram patterns irrespective of their position in the embedded text sequence.
- Embedding textual data is done by incorporating LSTM networks that provide comprehensive contextual and long-term relationship analysis of the data.
- CNNs and LSTMs are increasingly being used together as they combine the techniques for n-gram feature extraction that CNNs specialise in the sentence-level semantics that LSTMs capture^1,2^.

#### Random forest

Random Forest is used exclusively as a non-contextual baseline trained on TF-IDF bag-of-words features. No pretrained embeddings or semantic representations are used in the Random Forest experiments. The Random Forest classifier is trained solely on TF-IDF bag-of-words representations, without the use of pretrained embeddings or contextual representations. This configuration is intentionally employed as a non-contextual baseline to provide a direct comparison with the proposed CNN+LSTM model, which leverages pretrained semantic embeddings to capture contextual and sequential information in news articles.

Although transformer-based models such as BERT^3^ provide richer contextual representations through self-attention mechanisms, their high computational cost and memory requirements limit their practicality in resource-constrained environments. The CNN+LSTM architecture therefore, offers a favourable trade-off between contextual modelling capability, computational efficiency, and experimental reproducibility.

## Experimental setup

This section will discuss the context-aware news classification method. It will explain the datasets and evaluation metrics used. The objective is to design an evaluation exercise in support of the effective performance of the proposed models.

### Datasets utilized

For testing the effectiveness of the context-aware news classification approach, two publicly accessible benchmark datasets were used: the **Category Dataset V3** and the **AG News Corpus**. These datasets were selected due to their topical diversity, balanced category representation, and suitability for supervised learning tasks.

#### News category dataset V3

The **News Category Dataset (v3)**^25^ consists of a total of two hundred ten thousand two hundred ninety-four (210294) news headlines that the Huffington Post website published between the years 2012 and 2022. As shown in Tables 2 and 3, the dataset comprises news articles suitable for NLP, featuring high-quality text and detailed metadata. The attributes contained in the dataset are as follows for each of the news articles:

- **Category:** Classification of the article based on the sections it was published on (e.g., Politics, Entertainment, etc).
- **Headline:** The article’s title briefly explains the news.
- **Authors:** A list of the authors or, instead, other contributors.
- **Link:** Link to the article in the news site.
- **Short Description:** Brief introduction or article summary.
- **Date:** Last but not least is the news article’s publication date.

**Table 2:** Leading 10 categories within the dataset.

**News Categories**	**No of Articles**
Politics	35602
Wellness	17945
Entertainment	17362
Travel	9900
Style & Beauty	9814
Parenting	8791
Healthy Living	6694
Queer Voices	6347
Food & Drink	6340
Business	5992

**Table 3:** 10 categories appearing least in the dataset.

**News Categories**	**No of Articles**
Arts	1509
Environment	1444
Fifty	1401
Good News	1398
U.S. News	1377
Arts & Culture	1339
College	1144
Latino Voices	1130
Culture & Arts	1074
Education	1014

The inclusion of forty-two niche categories enhances the dataset’s versatility, making it suitable for a broader range of classification tasks. It also serves as a reference in engineering models that depend on meaning and context.

#### Dataset attributes and structure

**Fig. 2:**
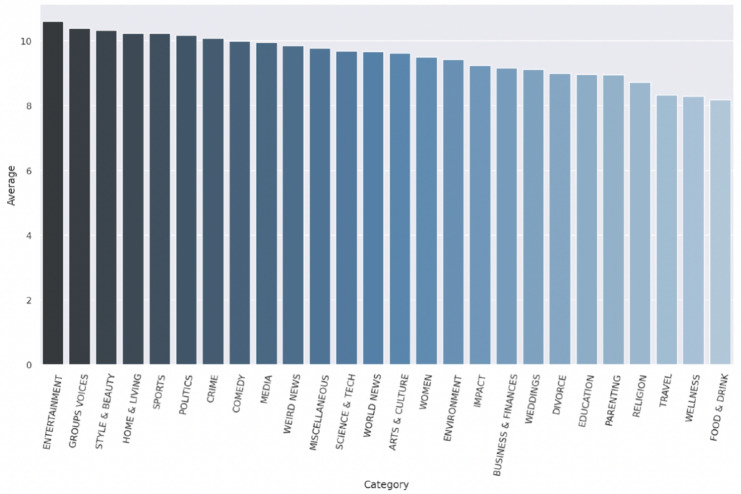
Visualisation of average headline lengths for articles categorised by topic.

**Fig. 3:**
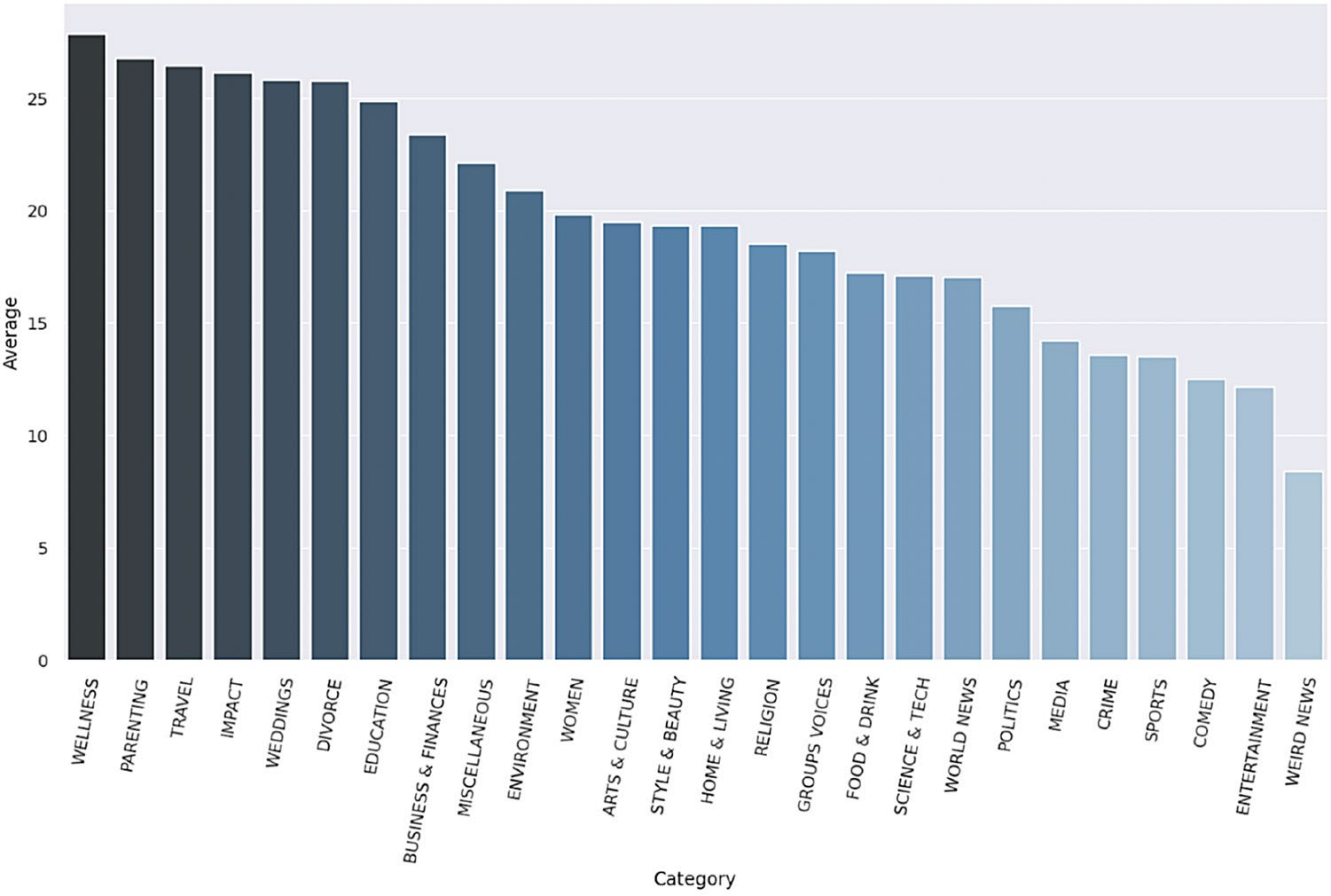
Visualisation of average abstract lengths for articles categorised by topic.

The provided dataset offers a very well-defined framework that allows for performing text classification tasks:

- **Category Distribution:** The dataset contains a wide range of classification categories from the most common, such as **Politics** (35,602 articles) and **Entertainment** (17,362 articles), to the less covered, such as Education (1,014 articles). This allows for the conduct of both global and particular classification attempts.
- **Headlines and Short Descriptions:** The dataset contains records of both the headline and the short description, enhancing semantic analysis. While headlines can be said to be minimalistic, short descriptions allow for better understanding and provide further details that aid in stronger NLP tasks. Figure 2 illustrates the mean length of headlines averaged across various categories of news articles. Figure 3 illustrates the mean length of abstracts averaged across multiple categories of news articles.

- **Temporal Coverage:** The data covers ten years, allowing for longitudinal studies and changes in journalistic practice over time.

#### Exploratory Data Analysis (EDA)

**Fig. 4:**
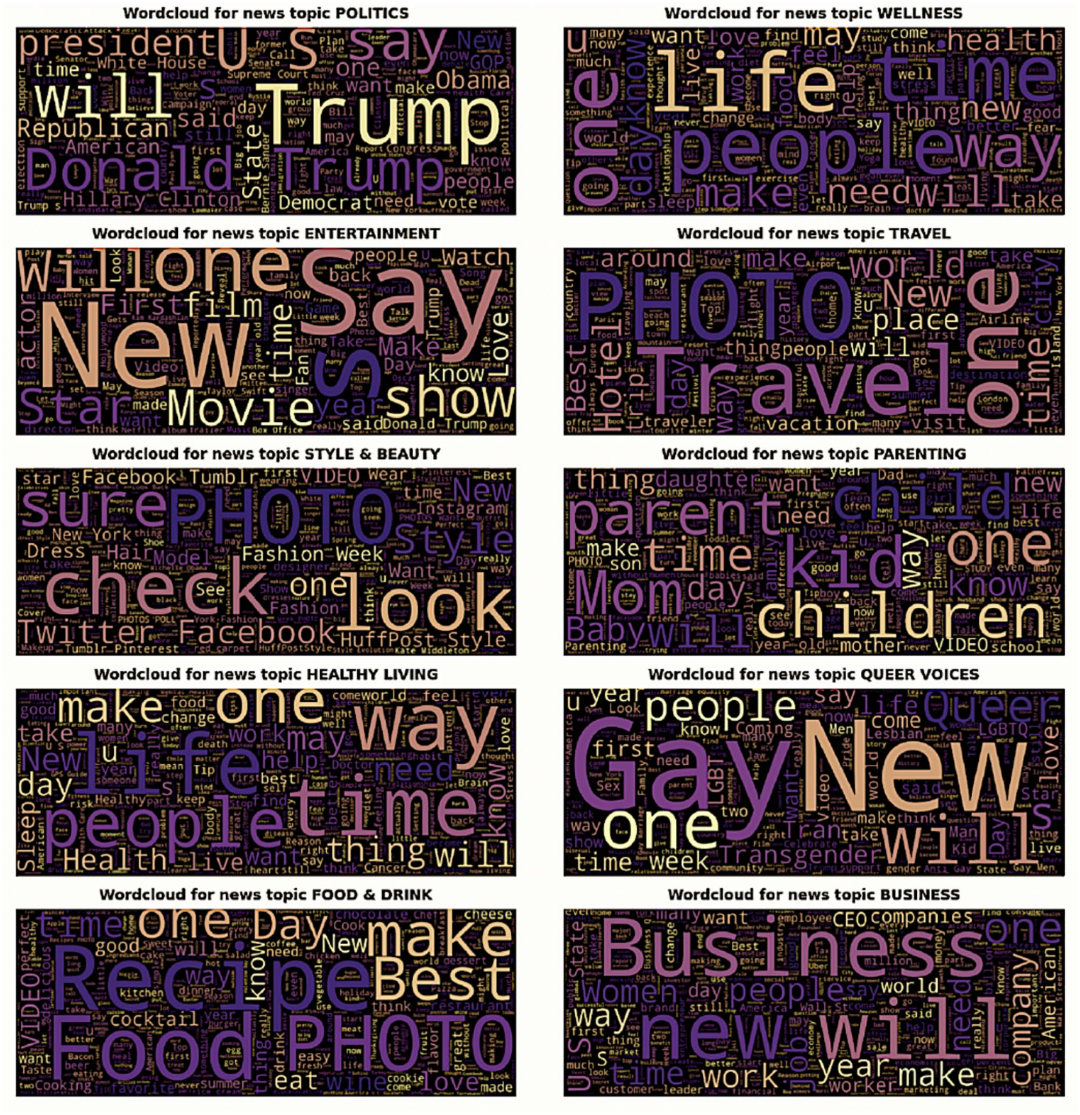
Word cloud visualisation of the top 10 news categories in the dataset.

To obtain some of the attributes of the dataset, a data exploration, or EDA as it is called, was carried out:

1. **Category Distribution:**
  The dataset consists mainly of categories such as Politics, Wellness, and Entertainment, which were some of the most popular in journalism during the collection period.
  On the other hand, categories such as Culture & Arts and Latino Voices are great examples of the variety that exists but do not have as many records.
2. **Headline and Description Length:**
  Across the categories, the average headline length is not very different, falling in the 7 to 10 word range.
  News on travel is sometimes elaborately explained in short descriptions. In contrast, sports news short descriptions are short, and separate entities can be found between them based on how long or short the category’s short descriptions tend to be.
3. **Word Clouds and Linguistic Features:**
  Linguistic tendencies observed in word clouds of top categories assist in semantic attribute identification for classification purposes. Figure 4 depicts a word cloud that illustrates some of the top news categories in the dataset, while the rest are the 10 most representative.

#### AG News Corpus

The **AG News Corpus** is a benchmark NLP dataset for news topic classification, comprising 120,000 training and 7,600 testing articles categorised into **World**, **Sports**, **Business**, and **Sci/Tech**. Created from the ComeToMyHead news aggregator and refined by Zhang et al.^26^ in 2015, it is widely used to evaluate both classical models like Naive Bayes and SVMs, and deep learning models such as CNNs and RNNs.

#### Data structure

Each entry in the AG News Corpus comprises three key components: a **class index** (an integer from 1 to 4) representing the news **category**, a title that serves as the headline summarising the article’s content, and a **description** providing a concise excerpt or detailed summary of the news. This structure enables flexible learning approaches, allowing the use of either the title, the description, or both in combination to achieve a deeper semantic understanding.

#### Implications for NLP modelling

Analysing headlines through the lenses of ML, an advanced DL model design looks to derive particular benefits from these averages concerning model length optimisation:

- **Input Size Adjustment:** The average sequence length helps determine padding, memory allocation, truncation, and even NP-hard optimisation in RNNs, GRUs, and Transformers.
- **Category-Aware Modelling :** Context-aware architectures that emphasise specific domain linguistics could exploit the discrepancies in text length per category.
- **Balanced Representation:** The consistent differences in description lengths suggest multi-input models (e.g., headline + description) to maintain a calibrated approach to categorically longer inputs and avert overfitting.

### cloud Environment: Google Colab (T4 GPU)

To train the GloVe-embedded CNN+LSTM model, a Jupyter Notebook-hosted service offered by Google Research, Google Colab, was used alongside the Random Forest classifier. Free NVIDIA GPU access provided by Colab facilitates rapid and large-scale training, as shown in Table 4 and Full reproducibility and training hyperparameters are summarised in Table 5.

**Table 4:** Cloud environment details.

**Component**	**Specification**
Cloud Platform	Google Colaboratory
Graphics Card (GPU)	NVIDIA Tesla T4 (16 GB GDDR6 VRAM, CUDA-enabled)
System RAM (Colab)	12.7 GB
GPU RAM	15.0 GB
Storage	112.6 GB
Environment Interface	Jupyter Notebook (hosted)
Operating System (Kernel)	Ubuntu 20.04 (Google-hosted VM)
Utilities	pandas, NumPy, matplotlib, scikit-learn

**Table 5:** Reproducibility and training configuration.

**Component**	**Value**
Input text	Title + Description (concatenated)
Max sequence length	100
Vocabulary size	200000
Embedding	GloVe (100-dim, pretrained, frozen)
Spatial dropout	0.2
CNN filters	128
CNN kernel size	5
Pooling	MaxPooling1D (pool size = 2)
LSTM hidden units	100
Dense layer units	100
Dropout	0.2
Optimizer	Adam
Loss function	Categorical Cross-Entropy
Learning rate	Default (Adam)
Batch size	32
Epochs	15
Random seed	42
Hardware	Google Colab (GPU-accelerated)

### Reproducibility and training details

The CNN+LSTM architecture, which combines spatial convolutional feature extraction with LSTM-based temporal sequence learning and the Random Forest classifier trained on TF-IDF bag-of-words features, serving as a non-contextual machine learning baseline, suggests that parallelised sandbox environments can accelerate prototyping and training cycles. This is why the cloud environment was so helpful.

### Evaluation metrics

The trained classifiers are evaluated on the test dataset using the following performance metrics:

- **Accuracy:** The proportion of total correct predictions to the overall number of instances is shown in Eq.1^27^.
  1 $$\begin{aligned} \text {Accuracy} = \frac{TP + TN}{TP + TN + FP + FN} \end{aligned}$$
- **Precision:** The proportion of true positive predictions to all positive predictions, indicating the classifier’s exactness, is shown in Eq.2^27^.
  2 $$\begin{aligned} \text {Precision} = \frac{TP }{TP + FP} \end{aligned}$$
- **Recall (Sensitivity):** The proportion of true positives to all actual positives, measuring the classifier’s completeness, is shown in Eq.3^27^.
  3 $$\begin{aligned} \text {Recall} = \frac{TP }{TP + FN} \end{aligned}$$
- **F1-Score:** The harmonic mean of precision and recall, providing a balanced assessment in scenarios with class imbalance shown in Eq.4^27^.
  4 $$\begin{aligned} F1\text {-}Score = 2 \cdot \frac{\text {Precision} \cdot \text {Recall}}{\text {Precision} + \text {Recall}} \end{aligned}$$

Accuracy, Precision, Recall, and F1-score were used to evaluate these models. These metrics comprehensively evaluate the classifier’s performance from multiple perspectives, enabling a fair comparison of model architectures. For imbalanced datasets, macro-averaged precision, recall, and F1-score are reported to ensure equal weighting of minority classes.

## Results analysis

This section presents the results of experiments on the proposed context-aware news classification framework. The goal is to determine how well performance is enhanced by incorporating contextual and semantic data into news classification engines. Results are analysed using benchmark evaluation metrics with multiple baselines and proposed models.

### News category dataset (Imbalance)

**Fig. 5:**
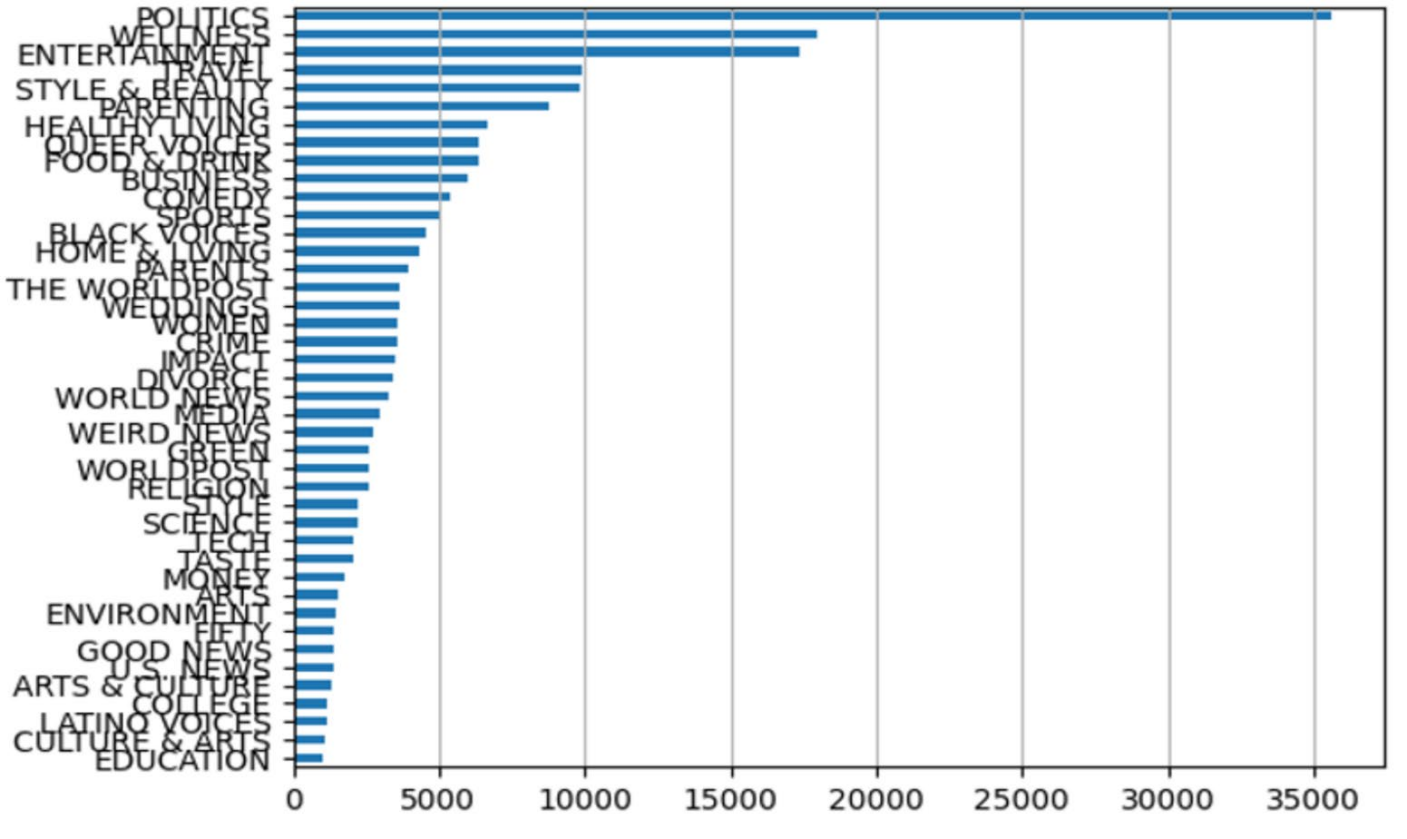
Class distribution in imbalanced news category dataset.

The research at hand utilises the News Category Dataset^25^, which includes more than 40 different categories of news. However, as imbalances are observable in the data shown in Fig. 5, some classes have far more samples than others. For instance, Politics, Wellness, and Entertainment categories contain over 20,000 samples while Education, Culture & Arts, and Latino Voices have less than 500 samples each. Such imbalances may pose a problem, as a bias toward learning is created when a model seeks to optimise for these frequently appearing categories, ultimately performing subpar on the rarely present categories. To analyse the influence of semantic context modelling on an imbalanced dataset, the following models were designed and assessed:

- **CNN+LSTM:** It is a multi-layered sequential architecture model incorporating convolutional layers for feature extraction and LSTM layers for sequence learning.
- **Random Forest:** Random Forest was trained using TF-IDF features with default tree-based aggregation.

**Fig. 6:**
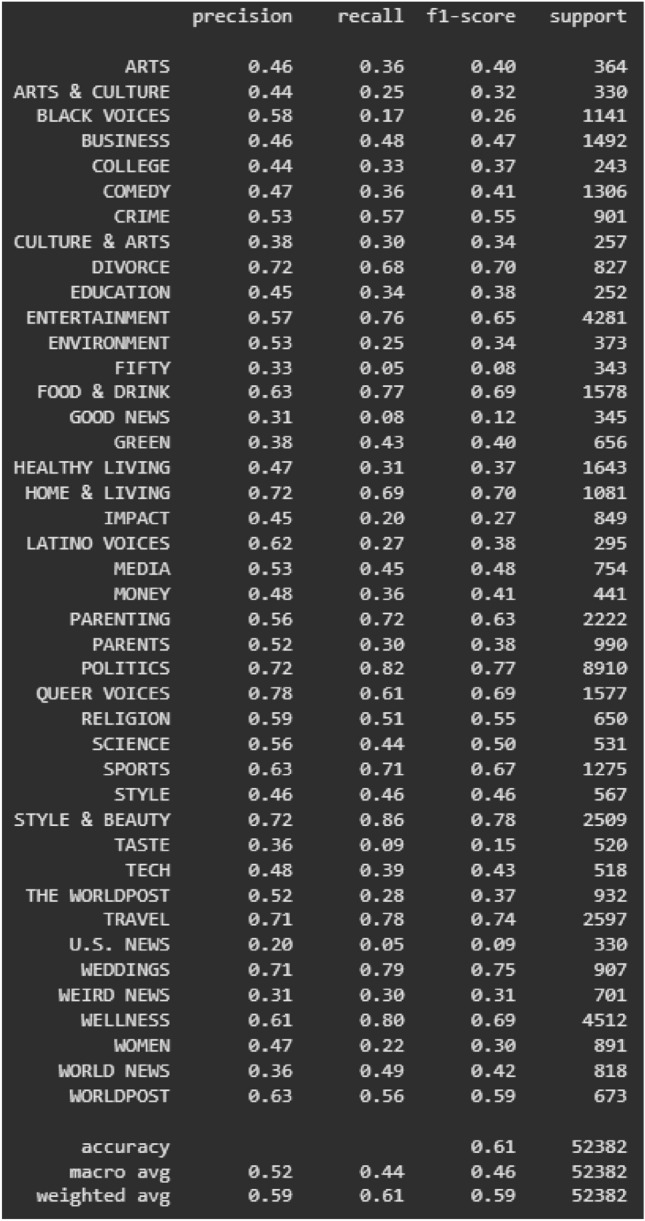
Confusion matrix for CNN+LSTM on the imbalanced news category dataset.

Both models are trained for 15 epochs. Assessment criteria encompass Accuracy, Precision, Recall, and F1-Score, which are calculated on a stratified validation subset. In addition to accuracy, macro-averaged F1-score is reported to account for minority class behaviour, and confusion matrices are provided for both imbalanced and balanced settings (Figs. 6 and 8).

#### CNN+LSTM

**Fig. 7:**
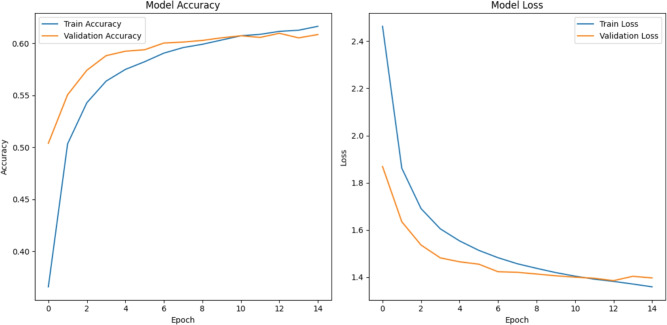
CNN+LSTM accuracy and loss over Epochs.

**Fig. 8:**
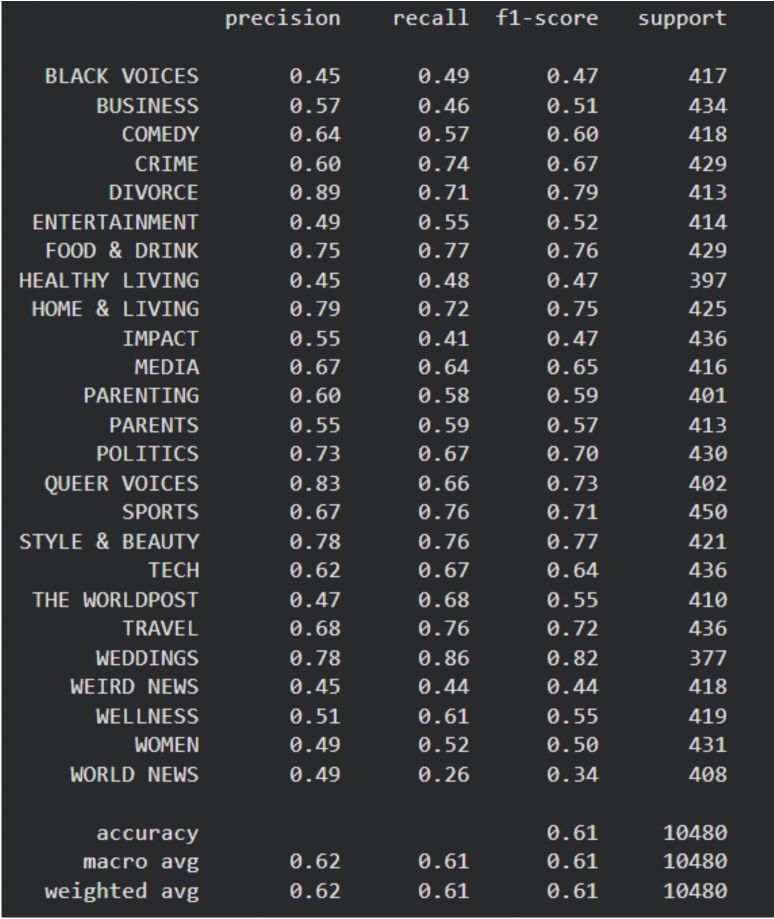
Confusion matrix for CNN+LSTM on the balanced news category dataset.

Similarly to most learners, the CNN+LSTM model, as depicted in Fig. 7, trains and continues to improve its validation accuracy. It achieves an approximate accuracy of 61% on the validation set. In addition to accuracy, macro-F1 was computed to better reflect the behaviour of minority classes in balanced settings, yielding a macro-F1 score of **0.61**. However, there is no improvement from epoch 10 onward. The validation loss reaches a plateau, signifying adequate generalisation, but may struggle to fully resolve long-range semantic dependencies. The Classification Report for CNN+LSTM on the balanced News Category Dataset, illustrating improved inter-class discrimination when class imbalance is mitigated in Fig. 8.

#### RandomForest

The Random Forest model showed weak performance on the imbalanced News Category Dataset. The model performs poorly in terms of precision and recall, particularly within minority classes. This indicates an inability to grasp the semantic context deeply, rendering the model inadequate for classifying complex texts. All Random Forest results reported in this study are obtained using TF-IDF bag-of-words features with default scikit-learn settings.

#### Comparative model performance

**Fig. 9:**
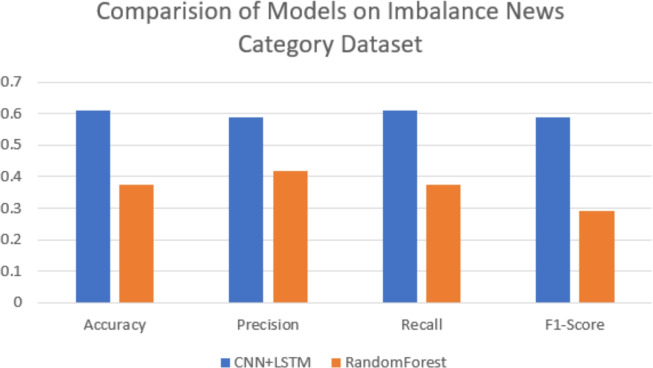
Comparison of CNN+LSTM and random forest on the imbalanced news category dataset across accuracy, precision, recall, and F1-score.

A quantitative comparison of all evaluated models on the imbalanced dataset is presented in Fig. 9. The results indicate that the CNN+LSTM model consistently outperforms the Random Forest baseline across all evaluation metrics, including accuracy, precision, recall, and F1-score. All Random Forest results are obtained using TF-IDF bag-of-words features. The inferior performance of Random Forest can be attributed to its reliance on surface-level lexical features, which limit its ability to capture semantic and contextual relationships in text, particularly under class-imbalanced conditions.

The confusion matrices in the Figs. 6 and 8 give more information in this regard. These matrices show that the CNN+LSTM model, when the class imbalance is addressed, demonstrates better intra-class separation, and this is reflected in the decrease of misclassification of semantically similar classes. Random Forest, on the other hand, demonstrates greater class confusion on the underrepresented classes, which reinforces its class imbalance sensitivity. Recent works point out that strategies for information-preserving and fairness-aware data augmentation can alleviate class imbalance without disrupting the underlying semantics^5,28,29^, suggesting that this could improve the performance of the models on the underrepresented classes.

### News category dataset (Balanced)

To address the class imbalance issue associated with the news article dataset, a Random Under-Sampling technique was applied during the data pre-processing stage. This process involved reducing the sample sizes of the majority classes to match those of the minority classes. In constructing a balanced subset of the News Category Dataset, **25 categories** were retained, each containing **2096 instances**. By this, an even distribution of classes was achieved so that the model could be trained without the overwhelming presence of high-frequency categories. The model was trained with the 25 categories to strike a balance between keeping enough topical diversity and having enough instances to enable stable training of the deep learning model.

Categories that had fewer than 1,000 instances, such as Latino Voices, College, and Fifty, were omitted in order to sidestep class-wise evaluations that were unreliable, and updates of the gradient that were unstable. The noise that such categories could add, along with the hindered convergence, and the misleading performance estimates, could be particularly problematic in deep learning. The categories that were described in the Table 6, ensure enough topical variety in the different areas of politics, lifestyle, culture, business, and social issues. It also ensures enough statistically meaningful and comparable evaluations across all classes.

**Fig. 10:**
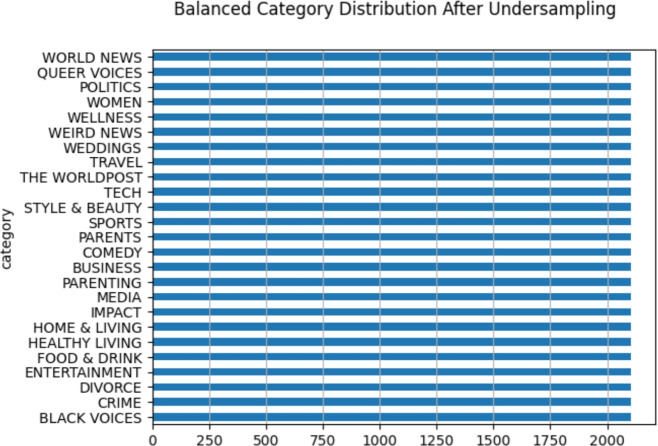
Class distribution in balanced news category dataset.

As illustrated in Fig. 10, the resulting dataset exhibits a uniformly homogeneous category distribution following the undersampling procedure. Consequently, each class was afforded an equal opportunity to contribute to model training, assessment, and evaluation, enabling unbiased and consistent performance analysis across all retained categories.

**Table 6:** Categories retained for balanced news category dataset.

**Category**	**Instances**
Politics	2096
Wellness	2096
Entertainment	2096
Business	2096
Travel	2096
Style & Beauty	2096
Food & Drink	2096
Parenting	2096
Healthy Living	2096
Queer Voices	2096
Sports	2096
World News	2096
Technology	2096
Science	2096
Education	2096
Culture	2096
Arts	2096
Media	2096
Environment	2096
Religion	2096
Crime	2096
Economy	2096
Health	2096
Lifestyle	2096
Society	2096

#### CNN+LSTM

**Fig. 11:**
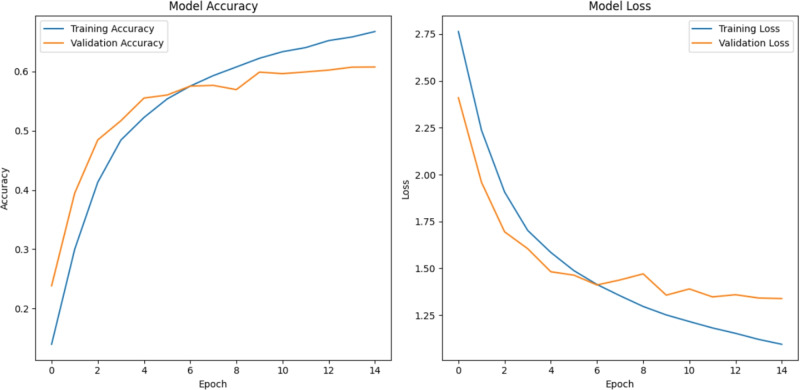
CNN+LSTM accuracy and loss over epochs on balanced news category dataset.

In contrast, Fig. 11 demonstrates that the CNN+LSTM model was able to achieve consistent improvement over the epochs. Nevertheless, the model was still only able to achieve a top performance of about 61% validation accuracy, even with balanced data.

#### RandomForest

The Random Forest model was the worst-performing model on the balanced News Category Dataset. This shows a lack of the model to sufficiently capture semantic context, indicating the model’s ineffectiveness on advanced-level text classification.

#### Comparative model performance

**Fig. 12:**
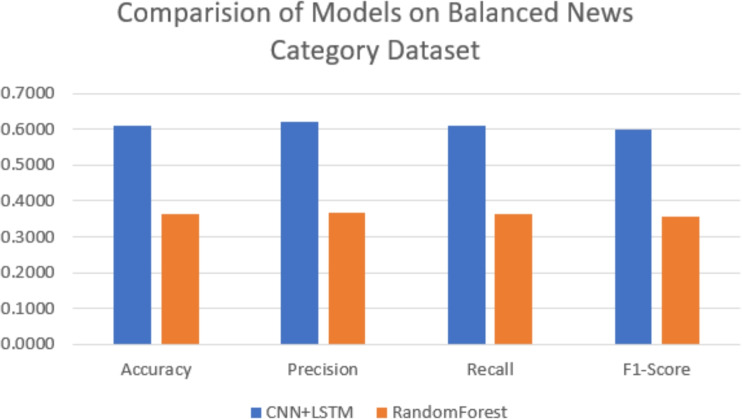
Performance comparison of classification models on balanced news category dataset.

The performance of both models is compared using a balanced dataset as illustrated in Fig. 12. CNN+LSTM significantly surpassed Random Forest.

### AG News Corpus

The AG News Corpus^26^ is a comprehensive and up-to-date dataset in terms of volume. Its breadth covers four classes **Business**, **Sci/Tech**, **Sports**, and **Worlds**, which describe themes found within news articles. The balanced nature of AG News Corpus allows researchers to evaluate model performance without the hurdles that class imbalance might introduce. The contention that stems from class imbalance, which might render models ineffectual, becomes irrelevant in measuring the AG News Corpus.

#### CNN+LSTM

**Fig. 13:**
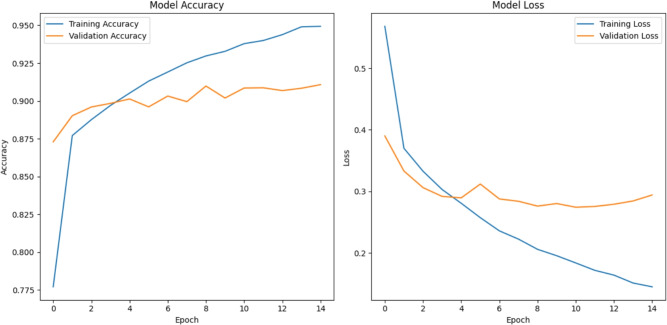
CNN+LSTM accuracy and loss over epochs on AG News Corpus.

CNN+LSTM showed roughly 91% validation accuracy as depicted in Fig. 13. Unlike the transformer-based models, CNN+LSTM does not achieve the same level of contextual adaptation as transformer-based models.

#### RandomForest

The Random Forest model achieved 80.77% accuracy on the AG News Corpus, with precision, recall, and F1 score close to 80.75%. Its performance was reasonable; however, the lack of deeper feature extraction yielded lower performance than other systems, such as RoBERTa+Bi-GRU, due to their use of transformers.

#### Comparative model performance

**Fig. 14:**
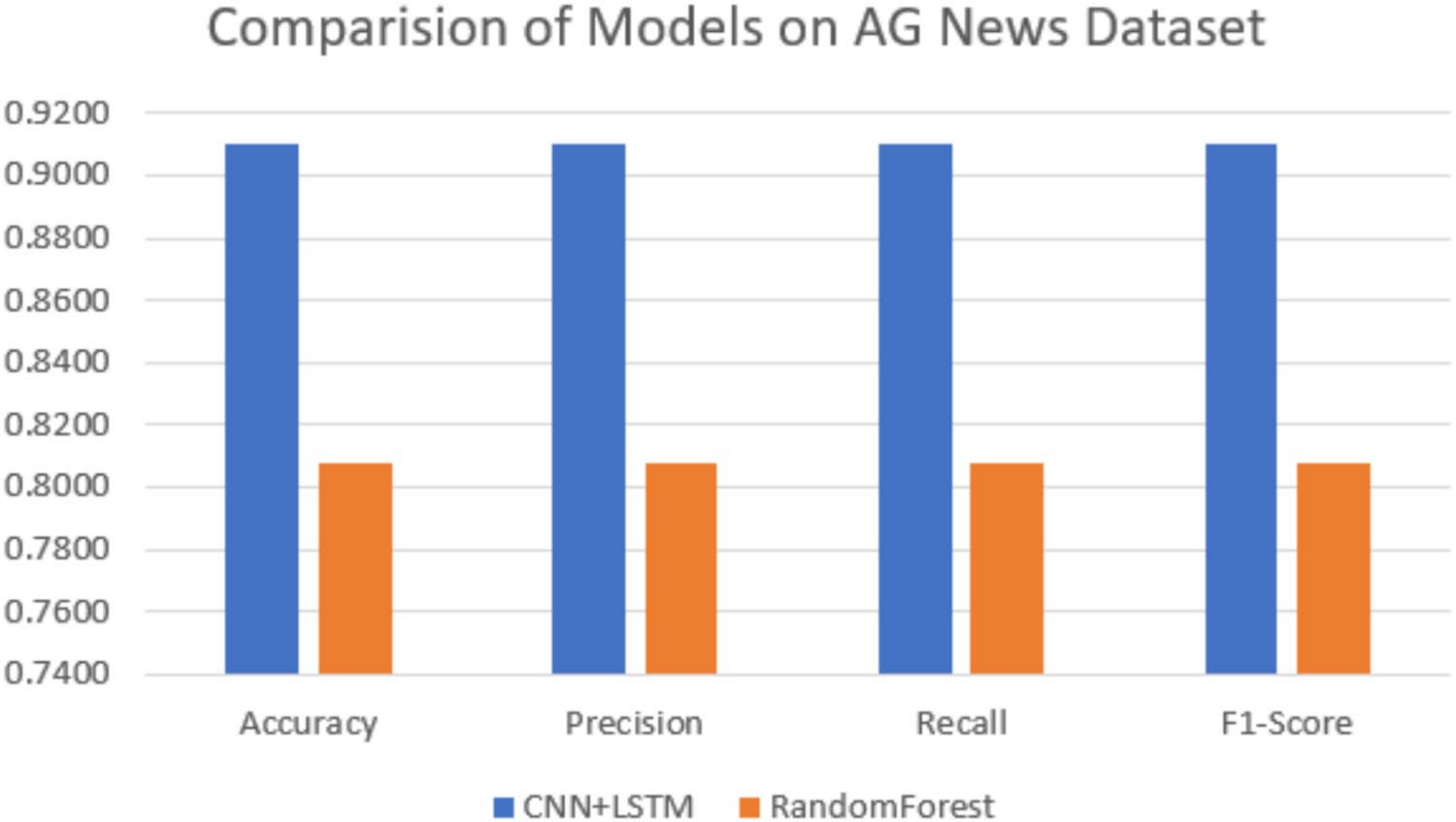
Performance comparison of classification models on AG News corpus.

The Comparison of all models is shown in Fig. 14. CNN+LSTM decisively outperformed Random Forest. All Random Forest results are obtained using TF-IDF bag-of-words features. This section presents the experimental results on both the CNN+LSTM utilising GloVe embeddings and a Random Forest classifier trained on TF-IDF bag-of-words features, serving as a non-contextual machine learning baseline. Based on evaluations of accuracy, precision, recall, and F1-score, the CNN+LSTM configuration consistently outperformed the Random Forest baseline. Its strong performance can be attributed to its ability to capture deep contextual and sequential dependencies within news texts.

While the Random Forest model provides some interpretability through feature importance analysis, the CNN+LSTM architecture operates as a distributed representation model and therefore offers limited inherent transparency. In this study, interpretability is addressed conceptually rather than through explicit explainability experiments. We acknowledge this as a limitation and note that incorporating attention mechanisms or post-hoc XAI techniques could provide deeper insight into model decision-making in future work, particularly for news and media applications where transparency is critical^5^. Overall, the experimental results indicate that contextual modelling significantly benefits balanced datasets, while class imbalance remains a limiting factor even for deep architectures.

## Conclusion

This study does not propose a novel model design, but rather provides a comprehensive empirical assessment of a hybrid CNN+LSTM architecture for news article categorisation. The results demonstrate that, despite its architectural simplicity, the CNN+LSTM framework remains a strong and efficient contextual baseline, particularly when evaluated across datasets with differing levels of imbalance and semantic complexity. The experiments were conducted on two benchmark datasets, the AG News Corpus, and the News Category Dataset V3. Across both datasets, the CNN+LSTM model with GloVe embeddings consistently outperformed a traditional Random Forest baseline in terms of accuracy, precision, recall, and F1-score. While the Random Forest model offered advantages in interpretability and lower computational cost, it proved limited in capturing deep semantic relationships and long-range contextual dependencies within news articles. Furthermore, the findings highlight the impact of class imbalance on model performance, with balanced data conditions yielding more reliable and consistent results for the CNN+LSTM framework. It is important to note that the reported accuracy exceeding 91% was achieved exclusively on the balanced AG News Corpus and should not be interpreted as representative of performance on fine-grained or imbalanced news categorisation tasks. This study is limited by its reliance on static word embeddings and the exclusion of transformer-based fine-tuning, which may yield superior performance on fine-grained or multilingual news classification tasks.

## Data Availability

The datasets generated and/or analysed during the current study are available in the Kaggle repository at: https://www.kaggle.com/datasets/mrameerhamza/news-category-datasets and https://www.kaggle.com/datasets/mrameerhamza/ag-news-topic-classification.
